# Not All Facial Droops Are Stroke: Miller Fisher Syndrome Presenting as a Stroke Mimic

**DOI:** 10.7759/cureus.9383

**Published:** 2020-07-25

**Authors:** Muhammad Hafiz Kamarul Bahrin, Syed Muhammad Ali Abidi, Kayteck Ling, Bhaskar Mukherjee

**Affiliations:** 1 Internal Medicine, Queen's Hospital Burton, Burton-on-Trent, GBR; 2 Medicine, Queen's Hospital Burton, Burton-on-Trent, GBR; 3 Geriatric Medicine, University Hospitals of Derby and Burton NHS Foundation Trust, Burton-on-Trent, GBR; 4 Geriatric Medicine, Queen's Hospital Burton, Burton-on-Trent, GBR

**Keywords:** miller fisher syndrome, guillain barre’s syndrome (gbs), stroke mimic

## Abstract

Miller Fisher syndrome (MFS) is a rare acquired neuropathy resulting from an acute infection and is believed to be a variant of Guillain-Barre syndrome (GBS). Its characteristic features are triads of ataxia, areflexia and ophthalmolegia, though involvement of cranial nerves is possible. Our case report describes a middle-aged man who presented as a potential stroke patient with left-sided facial droop, dysphagia and weakness. Upon in-depth clinical examination and basic investigations, stroke was deemed unlikely and clinical diagnosis of MFS was reached. This was further confirmed by the presence of anti-GQ1b antibody and anti-GT1a antibody in the serological study. Our patient was closely monitored with spirometry checks and only received supportive therapy throughout his treatment course until he achieved full clinical recovery. From this case we learnt that the clinical manifestations of MFS may vary depending on the presence of different types of autoantibodies. Similar to GBS, management of MFS is also largely supportive. Despite the widespread use of intravenous immunoglobulins with or without plasmapheresis to treat MFS, there is no conclusive evidence yet regarding prioritizing one treatment over another as the disease itself is self-limiting.

## Introduction

Miller Fisher syndrome (MFS) is a rare acquired polyneuropathy precipitated by an acute infection, more commonly respiratory than gastrointestinal (GI) or meningeal source, and it is recognized as a variant of Guillain-Barre' syndrome (GBS) [1]. The pathogenesis involves either demyelination or axonal injuries, and they are often self-limiting with good prognosis. This disease was first described by James Collier in 1932, presenting with triads of ataxia, areflexia and ophthalmoplegia [2]. Since then, more cases of MFS are being reported worldwide, involving additional neurological features such as bulbar and facial palsy. Unlike GBS, MFS often demonstrated ‘top down’ symptoms presentation in most cases [3]. It was postulated that the presence of different autoantibodies contributes to the symptomatology of the disease. This mixture of different neuropathic manifestations in MFS poses diagnostic challenge in an acute setting and is often confused with a posterior circulation stroke. Here, we describe a case of a man presenting initially as a ‘stroke alert’ patient to the local emergency department who was later diagnosed with MFS after thorough clinical judgement. He received only supportive care including nasogastric tube feeding throughout his admission. He made significant clinical recovery after about a week, with total resolution of symptoms after three months.

## Case presentation

We report a 65-year-old man who was brought into the emergency department at an early hour as a potential subacute stroke patient. He presented with left facial droop, slurring of speech and gait instability, resulting in collapse at home. At baseline, he was independently mobile, able to self-care and was able to walk up to a mile a day. He described that the symptoms were actually gradually worsening over a span of three to four days. He described that he has been feeling weak equally in all four limbs with occasional pins and needles sensation. On looking to left and right, he reported nausea secondary to double vision. He denied having headache, ear pain or discharge, fever or visual loss. He later admitted experiencing dysphagia for solids and liquids but they do not feel obstructed.

On further investigation, we noted that four weeks prior to this presentation, he was seen at the emergency department with one week history of sore throat. Diagnosis of tonsillitis was made and he was discharged with five days course of oral amoxicillin. The symptoms lasted for a week before it resolved but he felt as if he was losing his voice over the next three days. A tonsil swab sample was obtained during this attendance, which was later reported as normal.

His past medical history constituted only of hypertension, for which he takes amlodipine. His family history is unremarkable. In terms of his social history, he used to smoke tobacco cigarettes until 12 years ago. He smoked for about 30 pack-years. He drinks alcohol socially, amounting to about eight units of alcohol per week. He used to work as a bartender for 10 years.

His observation parameters on admission were stable with heart rate of 90 beats per minute, blood pressure of 130/70 mmHg, respiratory rate of 18 breaths per minute and temperature of 36.8 degree Celsius. Physical neurological examination demonstrated somewhat reduced power in upper limbs and lower limbs with Medical Research Council (MRC) grading of 4 to 5 out of 5. There was deep tendon areflexia in all four limbs. On walking, he demonstrated ataxic gait. There was also nystagmus in all horizontal gazes and dysmetria on finger-nose testing. On examining his face, we noted left-sided ptosis with left-sided lower motor neurone facial nerve palsy. On examining his eye movement, he demonstrated incomplete horizontal and vertical gaze palsy.

Following the local stroke protocol, CT imaging of the head was carried out and the result was unremarkable. In view of the complex set of presenting symptoms a few weeks apart from each other and normal CT, he was deemed unlikely to be suffering from a subacute stroke, and hence stroke treatment was not given. He was thought to have Bell’s palsy as a part of the diagnoses. Therefore, he was given 1 mg/kg of oral prednisolone as per the Trust guideline. He was admitted for observation and over the next few days, he was deemed unsafe to swallow. Decision was made to feed him through the nasogastric tube.

**Figure 1:**
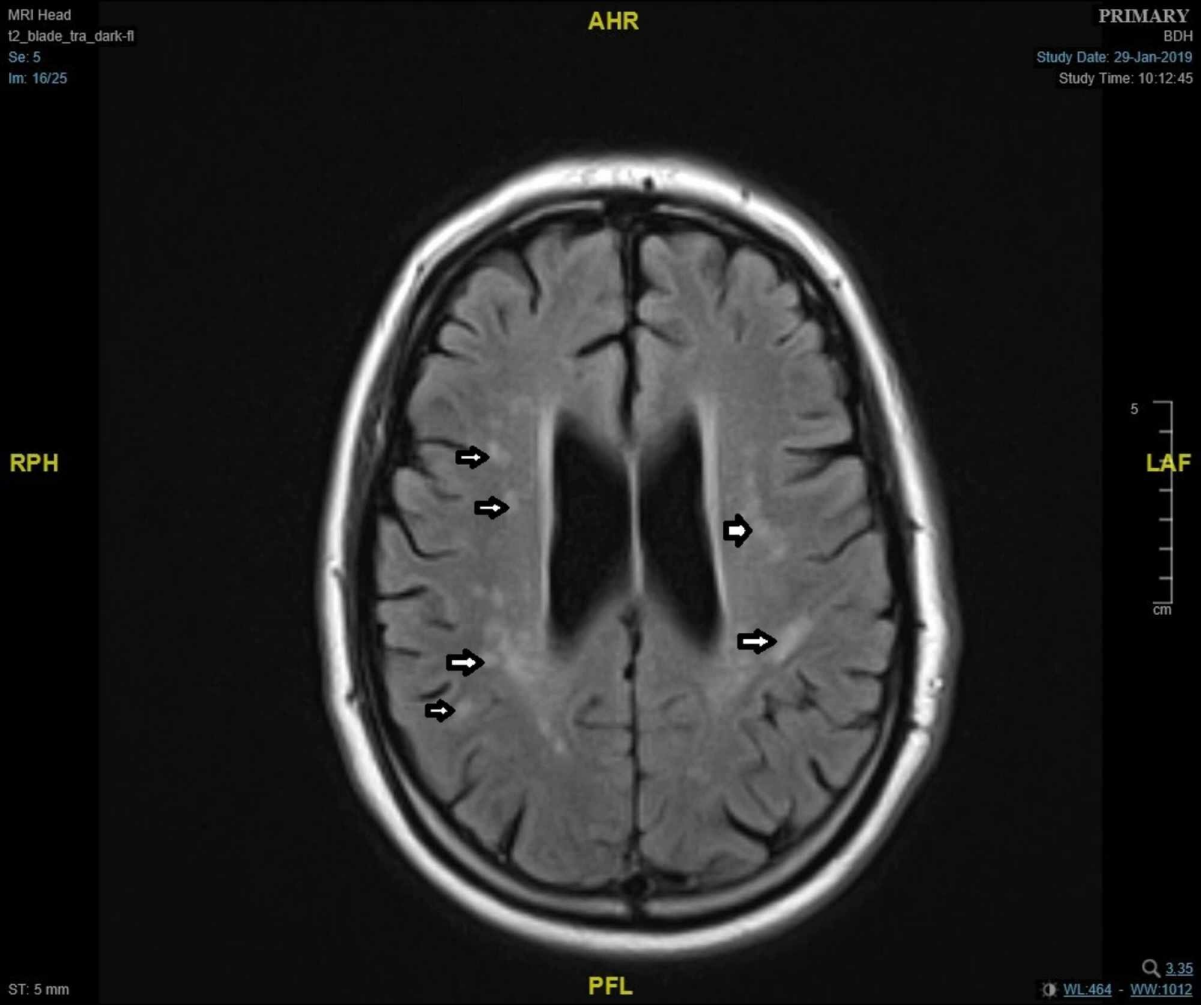
MRI demonstrating T2 hyperintense foci in cerebral white matter likely represent ischaemic gliosis secondary to small vessel disease. This raises suspicion of an acute demyelination.

Follow-up MRI during the admission showed T2 hyperintense foci in cerebral white matter representing ischaemic gliosis, which raises the suspicion of an underlying demyelinating disease (Figure 1). Upon discussion with the neurologist from the nearby tertiary centre, potential diagnosis of an acute inflammatory demyelinating disease secondary to recent upper respiratory tract infection such as MFS was made. Further laboratory investigations were carried out (Tables 1-3).

**Table 1:** Admission investigation results for full blood picture and electrolytes

Laboratory investigations	Result	Normal range
Full blood count (FBC)	133	140–170 g/L
White cell count (WCC)	10.1	4.5–11 x 10^9^ cells/L
Platelets	183	150–350 x 10^9^/L
Sodium (Na)	135	136–145 mmol/L
Potassium (K)	4.7	3.5–5 mmol/L
Adjusted calcium	2.38	2.2–2.6 mmol/L
Magnesium	0.76	0.62–0.99 mmol/L

**Table 2:** Cerebrospinal fluid (CSF) investigation results

Laboratory investigations	Result	Normal range
CSF protein	0.40	0.15–0.60 mg/ml
CFS glucose	3.73	2.50–4.40 mmol/L
CSF white blood cells	2	0/mm^3^

**Table 3:** Serological study results confirming Miller Fisher syndrome

Laboratory investigations	Result	Normal range
Anti-acetylcholine receptor antibody	0.00	0.00–0.20 nmol/L
Anti muscle-specific tyrosine kinase antibody (anti-MuSK)	0.00	≤0.02 nmol/L
Anti-GQ1b IgG antibody	Positive titre at 1/508	<1/500
Anti-GT1a IgG antibody	Positive titre at 1/508	<1/500

The positive titre of anti-GQ1b antibody confirmed the diagnosis of MFS. On top of that patient also showed cross reaction with anti-GT1a antibody, which we believe contributed to his characteristic cranial nerve involvement, which in this case, referring to the facial nerve and bulbar palsy.

Our patient was closely monitored in a medical ward with frequent spirometry check. His full vital capacity (FVC) was consistently over 2,500 cm^3^. He received only supportive care throughout, though we did plan to administer intravenous immunoglobulin in case of deterioration. He made significant physical improvement with residual ophthalmoplegia after one week of admission. After four days, his dysphagia resolved and the nasogastric tube was removed. As for the ophthalmoplegia, we provided him with prism lens. He underwent intensive rehabilitation thereafter and was followed up by the local neurologist on a three monthly basis.

## Discussion

MFS is an autoimmune disorder with wide range of neuropathies. It was discovered by Charles Miller Fisher in 1956. In his case series of three cases he differentiates MFS from GBS and characterized it as its variant [4]. MFS has an established strong male predominance (2:1) with highest prevalence in Japan and Taiwan of 25% and 19%, respectively [5]. It mostly affects people in their fifth decade but affects all age groups. MFS has also had gender preference with male affected more than females [4].

Rarity of MFS is recognized worldwide, with incidence of only one to two cases per one million population size across the globe [6]. Despite this, they have helped in understanding the process of autoimmune neuropathies. Molecular mimicry is the method of pathogenesis in MFS [5]. As with GBS, there is an evidence of preceding illness. Respiratory infections are noted in MFS with GI illness as the second most common cause. This is in contrast to GBS where in most cases GI illness precedes the immune reaction.

MFS is characterized by areflexia, ataxia and ophthalmoplegia without significant sensory loss [4]. It is a clinical diagnosis with availability of serum anti-GQ1b antibodies to confirm the diagnosis. These antibodies are found in 90% of cases [5]. The gangliosides are found in abundance in tissues around cranial nerves III, IV, and VI, and hence extraocular symptoms are most common presenting symptoms. The cerebrospinal fluid analysis in MFS usually shows albuminocytologic dissociation but a negative result should not rule out the diagnosis. Other antiganglioside antibodies such as anti-GT1a antibody may present in combination with anti-GQ1b antibody. The presence of anti-GT1a antibody, according to a study done by Koga, may determine disease manifestation involving ophthalmoparesis (70%), facial nerve palsy (57%) and bulbar palsy (57%) [7]. This exact pattern was observed in our patient.

Ataxia, areflexia and ophthalmoplegia can present in wide range of cases, such as GBS, cerebellar lesion, alcohol intoxication, encephalitis and Wernicke’s encephalopathy. In GBS, the most common presentation is limb weakness, whereas in MFS extraocular symptoms are predominant [4]. Cerebellar lesions present similarly to the MFS, but lack of lateralization in the latter helps it distinguished from cerebellar pathology [6]. Presentation of alcohol intoxication includes ataxia and balance problems, but the presence of other symptoms like slurred speech and impaired vision is also prominent.

Although MFS is a self-limiting disorder, disease-modifying therapies are not different from GBS. Intravenous immunoglobulin therapy and plasmapheresis are cornerstone treatments as recommended for GBS. Issues related to criterion for admission to intensive care unit or when to start on mechanical ventilation can arise even before initiation of specific treatment [6]. Due to self-limiting course of disease, there is limited evidence to which treatment is superior to other. A retrospective analysis on 92 cases of MFS showed that intravenous immunoglobulin therapy decreased the severity of ophthalmoplegia and ataxia, but there was no significant impact on overall outcome [6-8]. Complete symptom resolution for those affected by MFS or GBS is expected after six months in most cases; however, recovery tends to be better for MFS [2]. There is currently no established predictive factor of poor outcome in MFS unlike in GBS where hyponatraemia is associated with relatively poorer prognosis, believed to be due to developing syndrome of inappropriate antidiuretic hormone secretion (SIADH). 

## Conclusions

We learnt from our case study that MFS is a rare acquired neuropathy with incidence of one to two cases per million population worldwide. The presentations could sometimes be vague and may be a mixture of cranial nerve palsies depending on the presence of autoantibody types, hence posing a diagnostic challenge among inexperienced acute physicians. Often they could be confused with other acute neurological conditions such as stroke. Combinations of good clinical judgement, radiological and serological investigations are required to make a diagnosis of MFS. Neurophysiological studies will also help, although understandably this may not be easily accessible in most district general hospitals. Management of MFS is largely supportive as the disease is self-limiting. Its prognosis is good, and there is currently no established predictive factor of a poor outcome. Complete resolution of symptoms is expected after six months in most cases. Rapid progression of the disease, especially those that involve respiratory compromise, indicates intensive care unit (ICU) admission and the use of intravenous immunoglobulin or plasmapheresis or both.

## References

[1] Silk E, Golden C (2012). Miller Fisher syndrome. The Encyclopedia of Neuropsychological Disorders.

[2] Rocha Cabrero F, Morrison EH (2020). Miller Fisher Syndrome. https://www.ncbi.nlm.nih.gov/books/NBK507717/.

[3] Mori M, Kuwabara S, Fukutake T, Yuki N, Hattori T (2001). Clinical features and prognosis of Miller Fisher syndrome. Neurology.

[4] Fisher M (1956). An unusual variant of acute idiopathic polyneuritis (syndrome of ophthalmoplegia, ataxia and areflexia). N Engl J Med.

[5] Yepishin IV, Allison RZ, Kaminskas DA, Zagorski NM, Liow KK (2016). Miller Fisher syndrome: a case report highlighting heterogeneity of clinical features and focused differential diagnosis. Hawaii J Med Public Health.

[6] Gupta SK, Jha KK, Chalati MD, Alashi LT (2016). Miller Fisher syndrome. BMJ Case Rep.

[7] Koga M, Yoshino H, Morimatsu M, Yuki N (2002). Anti-GT1a IgG in Guillain-Barre syndrome. J Neurol Neurosurg Psychiatry.

[8] Yuki N (2009). Fisher syndrome and Bickerstaff brainstem encephalitis (Fisher-Bickerstaff syndrome). J Neuroimmunol.

